# Management of bilateral idiopathic healed sclerokeratouveitis with ciliary and intercalary staphyloma with complicated cataract and secondary glaucoma

**DOI:** 10.4103/0301-4738.67043

**Published:** 2010

**Authors:** Ruchi Goel, Lanalyn Thangkhiew, Usha Yadava, Sushil Kumar

**Affiliations:** Gurunanak Eye Center, Maulana Azad Medical College, New Delhi, India

Dear Editor,

We read the article by Parihar *et al*.[1] and agree with their conclusions and would like to share the course of a uveitic glaucoma with intercalary staphyloma which was managed well with phacoaspiration, posterior chamber intraocular lens (PCIOL), and Ahmed glaucoma valve (AGV) implantation. Uveitic glaucoma is challenging due to the early onset and the numerous mechanisms of pathogenesis including steroid-induced intraocular pressure (IOP) elevation.[2] Medical and surgical interventions though initially successful eventually fail due to fibrosis.[3,4]

A 32-year-old lady with recurrent episodes of pain and redness in both eyes (BE) for 3 years was referred to our center with uncontrolled high IOP in left eye. She was on topical timolol maleate 0.5%, brimonidine tartrate 0.15%, and dorzolamide 2% twice daily in BE since 6 months and complained of progressive diminution of vision, dark coloration of the sclera, and white opacities on the cornea for 1 year. There was not any history of ocular injury, fever, joint pain, skin lesions, or any other systemic illness.

**Figure 1:**
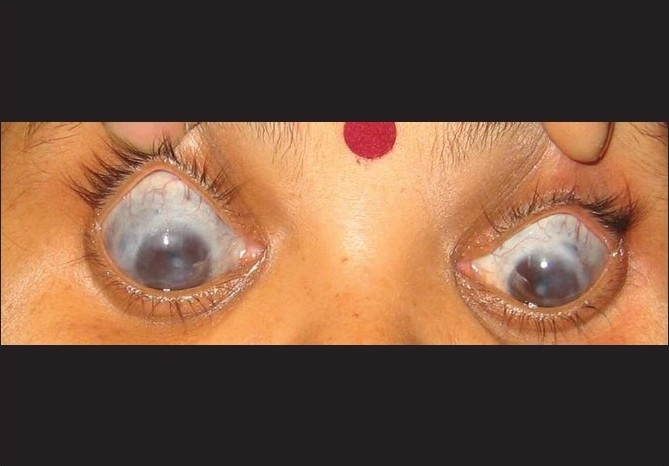
Both eyes showing healed sclerokeratouveitis with complicated cataract

On examination, vision in the right and left eye was 1/60 and 20/200, respectively. Both eyes had vascularized maculoleucomatous corneal opacities, normal anterior chamber depth, annular posterior synechiae, patent peripheral iridectomies, and cataract. She also had bilateral ciliary and intercalary staphyloma more in the right than the left eye. Tonopen reading in the right and left eye was 20 mmHg and 40 mmHg, respectively. Fundus and gonioscopy examination was not possible due to media opacities [Fig. 1].

B-scan of BE was anechoic. Complete blood counts, ESR, rheumatoid factor, cANCA (antinuclear cytoplasmic antibody), PANCA, antinuclear antibody, HLAB27, chest X-ray, and X-ray of sacroiliac joints were also normal.

**Figure 2:**
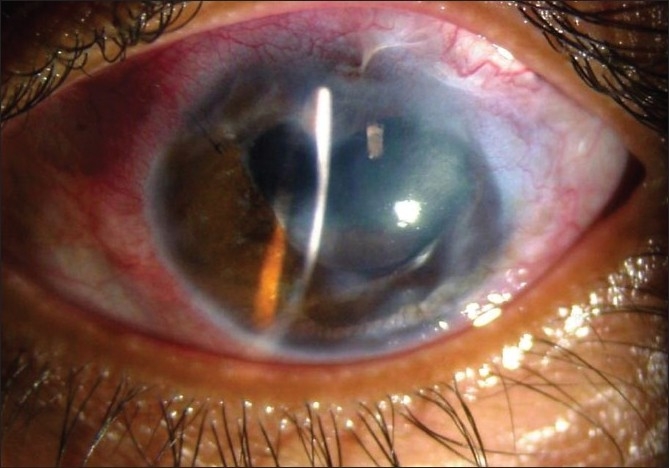
Postoperative appearance at 3 months

**Figure 3:**
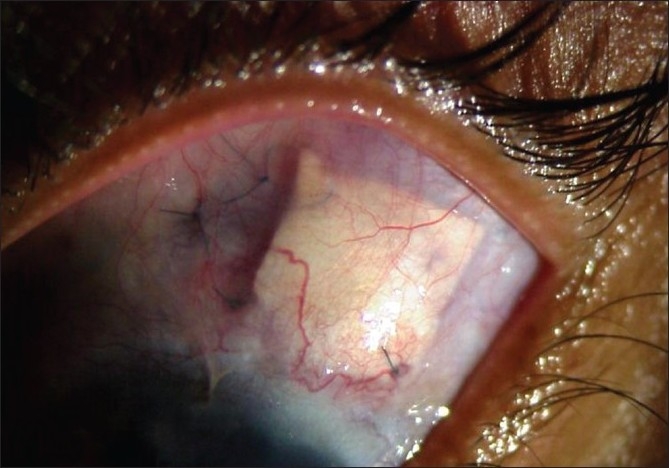
Cadaveric scleral graft covering the tube of the Ahmed glaucoma valve

We diagnosed this patient as bilateral idiopathic healed sclerokeratouveitis with ciliary and intercalary staphyloma with complicated cataract and secondary glaucoma. We planned phacoaspiration with PCIOL and AGV implantation as a single-stage procedure in the left eye under cover of oral prednisolone (1 mg/kg) tapered in 4 weeks. The plate of AGV was placed in the superotemporal quadrant and the tube was covered with the cadaveric scleral graft. Fixing the AGV tube and plate was an extremely uphill task due to thinness of sclera. Using iris retraction hooks, temporal phacoaspiration was performed and a multipiece acrylic foldable PCIOL was implanted in the bag. On the first postoperative day, left eye IOP was 17 mmHg and vision was 20/200. However, on the second day, there were a severe anterior chamber reaction, an exudative membrane over the IOL and iridectomy with an IOP of 22 mmHg and vision of finger counting at 1 m. We performed YAG membranectomy and pigment sweeping of the IOL on the fifth postoperative day. By the seventh day the IOP was 14 mmHg and vision improved to 20/63 and has remained in the lower teens in 2-year follow-up [Figs. 2 and 3].

We would like to conclude that glaucoma drainage device implantation is an appropriate primary surgical procedure in patients with refractory glaucoma with intercalary staphyloma and can be safely combined with phacoaspiration with PCIOL implantation.
